# Preliminary evaluation of the reliability, validity and feasibility of the arm activity measure – Thai version (ArmA-TH) in cerebrovascular patients with upper limb hemiplegia

**DOI:** 10.1186/s12955-018-0971-2

**Published:** 2018-07-16

**Authors:** Montana Buntragulpoontawee, Patreeya Euawongyarti, Tinakon Wongpakaran, Stephen Ashford, Somprartthana Rattanamanee, Jeeranan Khunachiva

**Affiliations:** 1 Department of Rehabilitation Medicine, Faculty of Medicine, Chiang Mai University, Chiang Mai, 50200 Thailand; 2 Department of Psychiatry, Faculty of Medicine, Chiang Mai University, Chiang Mai, 50200 Thailand; 3 Regional Hyper-acute Rehabilitation Unit London North West Healthcare NHS Trust & Department of Palliative Care, Policy and Rehabilitation, King’s College London, London, WC2R 2LS UK

**Keywords:** ArmA-TH, Validity, Reliability, Upper limb function, Outcome measure, Hemiplegia

## Abstract

**Background:**

Upper limb hemiplegia following cerebrovascular diseases can result in significant functional limitation. To assess such functional disturbance requires a comprehensive, valid and reliable tool. The Arm Activity Measure (ArmA) is a comprehensive, valid and reliable self-report questionnaire to assess real-life function for upper limb hemiplegia. However, it has never been translated for use in different languages. The purpose of this study is to translate and cross-culturally adapt the Arm Activity Measure (ArmA) questionnaire into a Thai version and to evaluate content validity, internal consistency and feasibility.

**Methods:**

The ArmA was translated and culturally adapted according to published cross-cultural adaptation guidelines resulting in the Thai version of ArmA (ArmA-TH). Forty Thai patients with upper limb hemiplegia resulting from cerebrovascular disorders participated in field-testing of the ArmA-TH. Its feasibility was evaluated. Content validity index for item (I-CVI) and score (S-CVI) were examined. Inter-rater reliability was evaluated by Gwet’s AC2. Internal consistency was measured using Cronbach’s alpha coefficient.

**Results:**

Forty patients (29 males, 11 females) with upper limb spasticity due to stroke or TBI were included. The average age of patients was 54.5 years (SD 15.0). Twenty-seven patients (67.5%) completed the questionnaire within 5 min or less, average time taken was 4.45 (1.73) min. For both subscales, patients reported the ArmA-TH to be relevant (85%) and easy to use (67.5%). More than 80% of patients found the passive subscale useful, almost 80% found the active subscale useful. Overall S-CVI was 0.83, S-CVI for passive and active function subscale was 0.79 and 0.86 respectively. The inter-rater reliability coefficients for ArmA-TH was 0.81. Cronbach’s alpha was 0.90 for the overall ArmA, 0.89 and 0.88 for the passive and active function subscales.

**Conclusions:**

The ArmA-TH was a feasible self-report questionnaire to assess hemiplegic upper limb function with good content validity, inter-rater reliability and internal consistency.

## Background

Hemiplegia is a common sequela following stroke or brain injury. Upper limb hemiplegia of varying degrees can result in significant functional limitations ranging from difficulty in caring the particular hemiplegic limb to increased difficulty for the limb to actively carry out a task [1, 2].

So far hemiplegic upper limb function can be assessed in many ways depending on the severity and which function to assess. Usually in severe patients whose limb becomes very spastic and has no or very little recovery, this limb is regarded as functionally “passive”, meaning the care must be provided for by caregiver or the opposite sound limb. For patients with moderate control or good recovery, the hemiplegic upper limb can assist or carry out some tasks or elements of tasks. This limb is considered functionally “active” [3].

In order to assess “real-life” hemiplegic upper limb function (day-to-day performance in the person’s normal environment), a number of self-reported outcome measures have been developed, for example, Arm Activity Measure (ArmA) [3–5], the Leeds Adult Spasticity Impact Scale (LASIS) [3, 5], Motor Activity Log (MAL) [3, 6], and Manual Ability Measure (ABILHAND) [3, 7, 8]. The ArmA was developed in 2013 by Ashford et al. and primarily intended to address both active and passive function comprehensively. It was designed to allow self-completion by the patient and/or carer enabling them to complete on their own whether at the clinic or at home and return by post. The psychometric properties of ArmA were tested and it was shown to have good validity, internal consistency, reliability and feasibility when used clinically [9].

However, presently in Thailand, hemiplegic upper limb function assessment was mainly limited to motor function assessment requiring rating by therapists, for example, Wolf Motor Function Test, Fugl-Meyer Test, and the Functional Test for Hemiplegic Upper Extremity in Persons with Hemiplegia – Thai Version (FTHUE-Thai version) [10, 11]. There is no self-reported outcome measure available in Thai, thus making objective “real-life” functional assessment difficult, especially after a treatment or research intervention. For these reasons, this study aimed to provide a translated and cross-culturally adapted Thai version of the ArmA and to examine its content validity, internal consistency and feasibility.

## Methods

### Population

The authors asked 40 patients with hemiplegic upper limb impairment due to stroke or traumatic brain injuries (TBI) from various places in Chiang Mai including outpatient rehabilitation and neurological clinic at Maharaj Nakorn Chiang Mai Hospital, Nakornping Hospital and The Northern Industrial Rehabilitation Center, to participate in the testing of the final ArmA-TH. All 40 patients gave written informed consent prior to completing the questionnaires. All were between 18 and 85 years of age with Thai mother tongue and graduated from at least elementary school with the ability to understand Thai communication in daily activities. All patients and caregivers (if present) were asked for informed consent before proceeding with the questionnaire. The patients’ characteristics included age, causes of upper limb hemiparesis, onset, education level, handedness, affected side, Modified Ashworth Scale (MAS), Brunnstorm stage of the upper limb.

#### The ArmA questionnaire

The ArmA questionnaire is a patient and/or carer-reported 20-item measure of difficulty in passive and active hemiparetic arm function. It consists of a seven-item passive function subscale, and 13-item active function subscale. Using a Likert scoring system between 0 (no difficulty) and 4 (unable to do task), the passive function subscale scores range from 0 (high function) to 28 and the active function subscale scores range from 0 (high function) to 52.

#### Translation and cross-cultural adaptation process

Following approval from the originator (Stephen Ashford - SA) the translation and cross-cultural adaptation of the ArmA questionnaire into Thai was conducted according to principles of the good practice report by the International Society for Pharmacoeconomics and Outcomes Research (ISPOR) Task Force for translation and cultural adaptation [12] along with the recommendations for the cross-cultural adaptation of health status measures by the American Academy of Orthopedic Surgeons [13].

Step 1: Forward Translation: Forward translation from English to Thai by two forward translators. One translator is a linguistic expert and another is a rehabilitation physician. Both translated the questionnaire independently resulting in two forward translations (Translation 1 - T1 and Translation 2 - T2).

Step 2: Reconciliation of T1 and T2: The authors and two forward translators discussed and resolved any discrepancies in the translations resulting in one common forward translation (T12).

Step 3: Back translation and review: Two native English speakers without medical background back translated the T12 independently resulting in two back translations namely BT1 and BT2. Both back translations were sent to be reviewed by the developer of the ArmA (SA) to ensure concept retention.

Step 4: Harmonization by authors and translators team: To ensure conceptual equivalence between the source and target language versions. The authors, forward translators and back translators, met and discussed discrepancies that arose between the different versions one item at a time until a consensus was reached. This resulted in the pre-final version of the Thai ArmA (ArmA-TH).

Step 5: Field testing and cognitive debriefing: The authors then invited 40 hemiparetic patients who were willing to participate in the field testing of the pre-final version of ArmA-TH. The purpose of this step was to evaluate how well the patients understood and responded to each item and allowed the authors to effectively detect minor errors. Afterwards, the authors met for discussion and identify any modifications necessary for improvement.

Step 6: Finalization and Proofreading: Lastly, the pre-final version was checked for any minor errors and corrected before the final version of ArmA-TH was approved for use among the target population.

The final ArmA-TH version was then qualitatively assessed by face-to-face interview with 10 volunteer patients to check patients’ understanding and responses to each item prior to actual research data collection.

#### Statistical analysis

The content validity was evaluated using Content Validity Index (CVI) [14]. In this study, 6 experts consisting of 2 neurologists and 4 physiatrists specialists were invited to rate the relevance of each item of the ArmA-TH. CVI was calculated as item CVI (I-CVI) and CVI for scale (S-CVI). I-CVI was the result of the number of experts answering in agreement (rate the item as relevant to very relevant) divided by the total number of experts. S-CVI could then be calculated by combining I-CVI of each item of the scale then divided by the total number of the combining items. The acceptable CVIs for items and scales were 0.79 and 0.89, respectively.

Inter-rater reliability was calculated using weighted Gwet’s AC2 by the method of ‘three raters or more’ with ordinal data because it is the most relevant to inter-rater agreement comparing to Fleiss’ Kappa, Krippendorff’s Alpha, and Brenann-Prediger [15] . This yielded chance-agreement coefficients. Benchmark scale of Landish and Koch was used to interpretation of the level of agreement as follows .8 to 1 (Almost Perfect), .6 to .8 (Substantial), .4 to .6 (Moderate), .2 to .4 (Fair), 0 to 2 (Slight), Less than 0 (Poor) [16, 17] . All data analyses were performed with IBM SPSS 22 (IBM Corp, 2013) and AgreeStat 2015.1 (Advanced Analytics, LLC, Gaithersburg, MD, USA).

Cronbach’s alpha coefficient was used to measure the internal consistency of the Thai version of ArmA with the acceptable value of > 0.7 The patients’ characteristics, ArmA scores, distribution of the scores, length of time to complete ArmA-TH and feasibility ratings were analyzed using descriptive statistics.

#### Feasibility questionnaire

The feasibility questionnaire in this study was applied from the original one by Ashford et al. to retain its purpose [9] . The questionnaire has 5 questions, and each asks about a) time to complete, b) relevance, c) usefulness of the active function section and e) ease of completion. Each is rated on a five-point Likert scale. The patients and carers completed the feasibility questionnaire on their own following ArmA-TH completion. Length of time taken to complete the ArmA-TH was also recorded.

## Results

### Translation and cross-cultural adaptation process

For the forward translation process, there were some minor differences between both forward translation versions (T1 and T2) for both the instruction part and for the details in each item, mainly because of grammatical and sentence structural difference between English and Thai. The translators also made sure that the Thai translation used simple and non-formal words to facilitate clear understanding and that back translation would result in close resemblance with the original ArmA. As soon as T1 and T2 were finished, a meeting was held to produce a common forward translation (T-12). Then, back translation from T-12 was carried out resulting in 2 back-translation versions (BT1 and BT2). Both BT1 and BT2 were sent to SA for comments. The questionnaire was rated as generally translated well and retained its original meaning, minor issues were identified. After expert committee meeting, some modifications of the instructions and items were made, for example, replacing “A” and “B” questionnaire section indicator with “1” and “2”, replacing “knife” with “spoon” in item 6 of Section B as Thai people rarely eat with a knife and fork. Another adjustment was to item 9 of section B “Dial a number on home phone”, the committee agreed to give details to 4 different types of phones as each require different functional capacity.

#### Evaluation of ArmA-TH

There were 40 patients in the questionnaire evaluation. The group composed of 29 men (72%) and 11 (27%) women, whose age ranged from 20 to 84 years with mean age of 54.9 years (SD = 15 years). Most patients (30 patients, 75%) graduated at elementary (18 patients) and high-school level (12 patients). Twenty-two patients had left hemiparesis (55%) while the rest had right hemiparesis (18 patients, 45%) as a result from hemorrhagic stroke (19 patients, 47.5%), ischemic stroke (16 patients, 40%) and lastly, traumatic brain injury (5 patients, 12.5%). Most had hemiparesis within the past 1–5 years (65%; see Table 1).

**Table 1:** Demographic characteristics of 40 patients completing the ArmA-TH questionnaire

Demographic characteristics	Number (%)
Age (year) – Mean (S.D.)	54.5 (15.0)
Sex Male	29 (72.5)
Female	11 (27.5)
Affected hand	
Left	22 (55.0)
Right	18 (45.0)
Both sides	0 (0.0)
Diagnosis	
Ischemic	16 (40.0)
Hemorrhagic	19 (47.5)
TBI	5 (12.5)
Education	
Non-education	1 (2.5)
Elementary school	18 (45.5)
High school	12 (30.0)
Vocational or high vocational certificate	5 (12.5)
Up to Bachelor of Arts	1 (2.5)
Not specified	3 (7.5)
Onset	
Less than a year	2 (5)
1–5 years	26 (65)
5–10 years	8 (20)
More than 10 years	4 (10)

### ArmA-TH scoring

The ArmA-TH passive score can range from 0 to 28, as for the active score, it can range from 0 to 52. The score of zero represents no difficulty and increasing score means increasing difficulty up to inability to do the activity. Results from 40 patients showed the ArmA-TH passive score ranged from 0 to 25 and active score from 2 to 43. The modal passive score was 10, as rated by 4 patients (10%). The modal active score was rather dispersed as 5, 10 and 15 were rated by 4 patients (10%). None of the patients score the maximum passive or active score.

### Feasibility

Time taken to complete the questionnaire varied from 2.4–10.6 min with average time of 4.5 (1.7) min. Twenty-seven patients (67.5%) completed the questionnaire within 5 min or less, almost all patients (95%) took under 10 min to complete. Seventeen patients (42.5%) indicated that they completed the questionnaire quickly to very quickly, while for 22 patients (55%) it took moderate amount of time. Ease of completion was rated as easy to very easy by 67.5% of patients. No patient rated the score as irrelevance and 85% rated the overall score as very relevant to most relevant. All patients rated the passive function subscale as moderately useful to very useful and over 95% rated the active function subscale as moderately to very useful.

### Content validity, inter-rater reliability and internal consistency

#### Content validity:

Patients who went through face-to-face interview responded positively to the questionnaire. When asked, the interviewers helped to clarify the active function part of the questionnaire that this part asked the ability to perform a task using both hands, not limited to only the paretic side. Only minor typing mistakes were found and corrected.

More than half of the ArmA-TH items (14 items) had very good I-CVI value ranging from 0.83–1.0. Both passive and active subscale had similarly high S-CVI values. As for the whole ArmA-TH questionnaire, S-CVI value was 0.83 (see Table 2).

**Table 2:** S-CVI and I-CVI of the ArmA-TH as rated by 6 experts

Item	Experts						
	1	2	3	4	5*	6*	CVI
Section A Caring for you affected arm (not using it in tasks or activities)							
1. Cleaning Palm	0	1	0	1	1	1	0.67
2. Cutting finger nails	1	1	1	0	1	1	0.83
3. Putting on a glove	1	0	1	0	1	1	0.67
4. Cleaning armpit	0	1	1	1	1	1	0.83
5. Putting arm through a sleeve	1	1	0	1	1	1	1.00
6. Put on a splint (If not used circle 0)	1	1	1	1	1	1	1.00
7. Positioning arm on a cushion or support in sitting (If not used circle 0)	1	0	0	1	1	1	0.67
Section B Using your affected arm to complete tasks or activities							
1. Do up buttons on clothing	1	1	1	1	1	1	1.00
2. Pick up a glass, bottle, or can	1	1	1	1	1	1	1.00
3. Use a key to unlock the door	1	1	1	1	1	1	1.00
4. Write on paper	1	0	0	1	1	1	0.67
5. Open a previously opened jar	1	1	1	1	0	1	0.83
6. Eat with a knife and fork	1	1	0	1	1	1	0.83
7. Hold an object still while using unaffected hand	1	1	1	0	0	0	0.50
8. Difficulty with balance when walking due to your arm	1	0	1	1	0	1	0.67
9. Dial a number on home phone	1	1	1	0	1	1	0.83
10. Tuck in your shirt	1	1	1	1	1	1	1.00
11. Comb or brush your hair	1	1	1	1	1	1	1.00
12. Brush your teeth	1	1	1	1	1	1	1.00
13. Drink from a cup or mug	1	1	0	1	1	1	0.83

Note: Section A contains 7 passive function items and Section B contains 13 active passive items

#### Inter-rater reliability:

The inter-rater reliability co-efficient of all ArmA items was 0.81, which was.

“Substantial” according to Table 3 showing the coefficients for inter-rater reliability using Gwet’s AC2.

**Table 3:** Benchmarking Gwet’s AC2 Weighted Agreement Coefficients using Cumulative Membership Probabilities

	Method	Coefficient		
		Arm-A	Passive	Active
Benchmark Scale	Gwet’s AC_2_	0.81	0.81	0.82
	Percent agreement	0.85	0.87	0.86
Scale (Item7 removed)	Gwet’s AC2	0.88	0.81	0.90
	Percent agreement	0.91	0.87	0.92

For all items of ArmA, it yielded the coefficients of 0.81 suggesting ‘substantial’ according to the Landis-Koch benchmark scale. The AC2 increased from 0.82 to 0.90 for active subscale after the item 7 has been removed.

#### Internal consistency:

The Cronbach’s alpha for ArmA-TH was 0.90. For the passive subscale Cronbach’s alpha was 0.89 and 0.88 for the active subscale.

## Discussion

To the authors’ knowledge, the ArmA is the only published self-report questionnaire aiming to comprehensively measure both passive and active function of the paretic upper limb in real life situation [9]. Concerning the need for a similar outcome measure among Thai patients, the authors obtained permission to translate and cross-culturally adapted the ArmA according to ISPOR guidelines [12] resulting in ArmA-TH. To ascertain that the ArmA-TH retained its original concept, the process was periodically reviewed by the originator of the English language version with comments provided, all comments were carefully discussed to reach a consensus resulting in the ArmA-TH. The adjustments made during the cross-cultural adaptation process were related to cultural differences, for example in Thailand, utensils for eating are spoon and fork instead of knife and fork as meals usually consist of rice and side dishes. Also, the committee agreed on adding the telephone type details because of its great variety in Thailand. When answering the questionnaires, most could do so quickly to very quickly. More than half of the patients could complete the questionnaire within 5 min. The short duration taken is encouraging for its use in future real-life setting. This finding is similar to the original ArmA in English [9] .

Here we tested content validity again in order to make sure the concept was well-retained and well-adapted to Thai cultural context. Interestingly, more than half of ArmA-TH 20 items had very good I-CVI range of 0.83–1.00, 7 items had moderate I-CVI of 0.67. One particular item however, item 7 of active function subscale had I-CVI of 0.50, showing that 3, consisting of 2 neurologists and 1 rehabilitation specialist, of 6 experts rated the item as irrelevant. This item asks the patient to rate difficulty to “hold an object still while using unaffected hand”. This finding is in marked contrast to the findings in the original ArmA development, where this item was rated as very relevant by patients, care-givers and clinicians (Physiotherapist, Occupational Therapist and Rehabilitation Medicine). There are three possible explanations for this; 1 a misunderstanding of the concept represented in the question by clinicians; 2 differences perception between patients and clinicians in what the priority areas are; or 3 a simple misunderstanding of the language used related to the translation. Looking back at the translation process, this item was back translated as “able to use disabled hand when using the good hand” by back translator 1 and “hold something steady while using your good hand” by back translator 2, the latter was confirmed as closer to original when reviewed (SA). Therefore, language difference may play a role. Back translation 1, “Able to use disabled hand when using the good hand” was different from back translation 2 and also from the original “Hold an object still while using unaffected hand”, so there was a possibility that clinicians might have a different perception of the item rather than the true meaning. Since the ArmA has never been translated to other languages before, the authors cannot compare this finding with other translations, or even the original ArmA because its content validity was not addressed by CVI method. For the S-CVI, the authors chose to calculate by averaging all items I-CVIs to focus on the questionnaire item relevance to the underlying construct [14]. The S-CVI for the active subscale is slightly lower than the passive subscale and the overall ArmA-TH scale because item 7 in the active subscale had I-CVI of 0.5.

While CVI is aimed at examining how each item is valid for the whole scale, it assesses the extent to which raters consistently distinguish between different responses [17]. Our results show that they are reliable raters. The inter-rater reliability results were “almost perfect” agreement for both subscales and the overall ArmA-TH.

Even though the number of patients were limited to 40, the Internal consistency as measured by Cronbach’s alpha still showed high internal consistency in both subscales, 0.89 for passive function, 0.88 for active function and 0.90 for the whole ArmA-TH. The values are comparable to the original ArmA questionnaire which showed Cronbach’s alpha of 0.85 for passive scale and 0.96 for active scale.

However, confirmatory factor analysis using larger and sufficient sample size will be carried out in our future work. In addition, an alternative reliability analysis such as ordinal alpha or omega will be considered with these ordinal data.

Lastly, this study was a preliminary study to demonstrate the translation and cross-cultural adaptation of the ArmA-TH and to examine the basic measurement properties of the ArmA-TH.

More detailed study on other important aspects of psychometric properties, for example, construct validity, test-retest reliability and responsiveness should be conducted.

## Conclusions

The ArmA-TH is the first self-reported questionnaire in Thai to assess function for upper limb hemiplegia. This study not only showed its comprehensiveness, measuring both “passive” and “active” functional aspects, but also demonstrated its good content validity and high internal consistency. Moreover, it took a short time to complete for patients and carers and was well-accepted, making it an objective and feasible outcome measure tool to assess hemiparetic upper limb real-life function in both clinical and research settings.

## Abbreviations

ABILHAND: Manual Ability Measure
ArmA: Arm Activity Measure
ArmA-TH: Arm Activity Measure Thai Version
CVI: Content Validity Index
FTHUE-Thai version: Functional Test for Hemiplegic Upper Extremity in Persons with Hemiplegia – Thai Version
I-CVI: item Content Validity
ISPOR: International Society For Pharmacoeconomics and Outcomes Research
LASIS: Leeds Adult Spasticity Impact Scale
MAL: Motor Activity Log
MAS: Modified Ashworth Scale
S-CVI: Content Validity for Scale
